# Timing and Location of Medical Emergency Team Activation Is Associated with Seriousness of Outcome: An Observational Study in a Tertiary Care Hospital

**DOI:** 10.1371/journal.pone.0168729

**Published:** 2016-12-28

**Authors:** Takeo Kurita, Taka-aki Nakada, Rui Kawaguchi, Koichiro Shinozaki, Ryuzo Abe, Shigeto Oda

**Affiliations:** 1 Department of Emergency and Critical Care Medicine, Chiba University Graduate School of Medicine, Chiba, Japan; 2 The Feinstein Institute for Medical Research, Manhasset, New York, United States of America; Osaka University Graduate School of Medicine, JAPAN

## Abstract

**Purpose:**

The medical emergency team (MET) can be activated anytime and anywhere in a hospital. We hypothesized the timing and location of MET activation are associated with seriousness of outcome.

**Materials and Methods:**

We tested for an association of clinical outcomes with timing and location using a university hospital cohort in Japan (n = 328). The primary outcome was short-term serious outcome (unplanned ICU admission after MET activation or death at scene).

**Results:**

Patients for whom the MET was activated in the evening or night-time had significantly higher rates of short-term serious outcome than those for whom it was activated during the daytime (vs. evening: adjusted OR = 2. 53, 95% CI = 1.24–5.13, *P* = 0.010; night-time: adjusted OR = 2.45, 95% CI = 1.09–5.50, *P* = 0.030). Patients for whom the MET was activated in public space had decreased short-term serious outcome compared to medical spaces (public space: adjusted OR = 0.19, 95% CI = 0.07–0.54, *P* = 0.0017). Night-time (vs. daytime) and medical space (vs. public space) were significantly associated with higher risks of unexpected cardiac arrest and 28-day mortality.

**Conclusions:**

Patients for whom the MET was activated in the evening/night-time, or in medical space, had a higher rate of short-term serious outcomes. Taking measures against these risk factors may improve MET performance.

## Introduction

The concept of a medical emergency team (MET) has been implemented worldwide to rapidly respond to patients in a deteriorating condition, as well as to prevent in-hospital cardiac arrest, unexpected intensive care unit (ICU) transfers, and mortality [1–3]. The medical emergency team (MET) can be activated anytime and anywhere in a hospital, not always in general wards but also in the areas for examination, treatment and outpatients as well as public space [4, 5]. Therefore, in a hospital, any member of a staff might encounter a patient whose life is in danger, and all can improve outcomes by recognizing the situation early and activating the MET.

Medical resources may differ between the various departments in a hospital; the baseline characteristics of patients in a deteriorating condition may also vary. These may in turn lead to variation among departments in the frequency of MET activation, as well as in subsequent clinical outcomes. However, evidence is limited to a few investigations comparing this frequency between monitored and unmonitored units [6, 7]; it remains unclear whether the location of MET activation is associated with frequency or clinical outcome.

Timing is the other important factor in the frequency and clinical outcomes of MET activation—temporal differences in the frequency of MET activation have been reported. For instance, MET activation is more common on Mondays [8], in the morning [9], during the daytime [6, 10] or work hours [8], during routine nursing observation, and during handover [10] or the routine overnight nursing observation time [11]. Despite this accumulating evidence for temporal differences in the frequency of MET activation, it remains unknown whether the timing of MET activation is associated with clinical outcome.

If investigators elucidate the relationships between timing/location and clinical outcome, healthcare providers at the scene may be more aware of at-risk patients. This would improve MET-based care through early recognition and activation.

We hypothesized that the timing and location of MET activation are associated with seriousness of outcome. Therefore, using a university hospital cohort in Japan, we investigated whether clinical outcome after MET activation differed by timing and location. The primary outcome variable was short-term serious outcome (unplanned ICU admission or death at scene). The secondary outcome variables were unexpected cardiac arrest upon receipt of MET call and 28-day mortality after MET activation.

## Materials and Methods

### Medical emergency team

We conducted an observational study at Chiba University Hospital, Japan. At the time of the study, the hospital had a total of 835 beds, including 22 ICU beds, for medical, surgical, and cardiovascular patients. There were 739 physicians treating an average of 2,064 outpatients and 759 inpatients daily in 2014. Prior to the implementation of our MET into the hospital, all hospital workers had been informed about inclusion and exclusion criteria described below. The notification was completed three months in advance of the MET implementation. The hospital workers were communicated orally and/or by document (the brochure of hospital manual) in order that everyone working in the hospital knows how to activate our MET.

The MET consisted of physicians from the department of Emergency and Critical Care Medicine, as well as nurses/clinical engineers from ICU. The MET can be activated for anyone within the hospital grounds: inpatients, outpatients, visitors, hospital staff, and vendors who require an emergency procedure. All hospital staff, including physicians, nurses, clinical engineers, medical assistants, and technologists can activate the MET in response to the following pre-defined MET activation criteria: obstructed airway, abnormal breathing, circulatory failure, disturbance of consciousness, trauma, unexpected cardiac arrest, a risk of any of these conditions, and any other deteriorating condition that the discoverer considers worthy of MET activation; exclusion criterion is: patients presenting the condition as a consequence of considerable terminal illness. There are no criteria based on the absolute value of vital signs (such as blood pressure, lactate levels, respiratory rate etc.).

### Subjects

We screened all patients for whom MET activation was requested between February 2012 and January 2015 (n = 336), excluding eight patients who had data missing. Thus, 328 patients were included in the analysis. The Institutional Review Board at the Chiba University Graduate School of Medicine approved the current study. The review board waived the need for written informed consent.

### Definition and data collection

In the present study, short-term serious outcome was defined as unplanned ICU admission after MET implementation, or death at the scene where MET care was initially implemented [1] [12]. Patients who had short-term serious outcome were assigned to the “Serious”, while the remaining patients were assigned to the “Non-serious” group. Similarly, patients who suffered unexpected cardiac arrest upon receipt of the MET call were assigned to the “Cardiac arrest” group, and the remaining patients were assigned to the “Non-cardiac arrest” group.

Furthermore, patients were categorized according to the time of the MET call: daytime (9:00–16:59; regular business hours of the hospital), evening (17:00–0:59) and night-time (1:00–8:59). We defined Saturdays and Sundays as “weekend”, and the remaining days as “weekdays”. We defined off-hours as during the evening, night-time, or weekend. In the same way, patients were categorized according to the location of the incident: “medical space”, “general wards”, and “public space”. “Medical space” comprised the outpatients’ department, examination rooms (blood collecting room, radiographic examination room, and physiological laboratory), and treatment rooms (operating room, endovascular room, dialysis room, and delivery room). “Public space” comprised the hall, stairs, lounge, and hospital parking lot. Sepsis was defined using the 2001 international sepsis definitions [13].

Data was prospectively collected by both the requester and the MET, who completed designated MET data forms, which were then independently checked by data managers, who created the data set for the analysis. We made a report for each case after MET was activated regardless of patients’ severity or managements by MET. There are no data lost once our MET is activated.

### Statistical analysis

The primary outcome variable was short-term serious outcome (unplanned ICU admission after MET intervention or death at scene). The secondary outcome variables were unexpected cardiac arrest upon receipt of the MET call and 28-day mortality.

Differences among timing of MET calls in terms of the probability of short-term serious outcome, unexpected cardiac arrest, and 28-day mortality (daytime vs. evening vs. night-time; weekday vs. weekend) were tested using the chi-square test. Differences in baseline characteristics and clinical outcomes between the Serious and Non-serious groups were analyzed using the chi-square test (for categorical data) or the Mann–Whitney *U* test (for continuous data).

As the primary analysis, we used multivariate logistic regression to test for differences in short-term serious outcome by timing (daytime vs. evening vs. night-time; weekday vs. weekend) and location (medical space vs. general ward vs. public area). Such an analysis involved adjustment for potential confounding factors based on biological plausibility and previous studies [14–19] using a cut-off *P*-value < 0.1 in the univariate analysis of baseline characteristics (variables: age, patient status [inpatient or not], cardiovascular diagnosis, medical history [postoperative, post ICU discharge, sepsis on MET activation], chronic heart failure, and chronic kidney disease).

To evaluate the effect of timing and location on the secondary outcomes, we used multivariate logistic regression analysis, which adjusts for confounding factors (as listed in the primary analysis). Two-tailed *P-*values of < 0.05 were considered significant. Odds ratios (ORs) and 95% confidence intervals (CIs) were shown. Analyses were performed using SPSS (SPSS, version 20, Armonk, NY, USA) statistical software.

## Results

**Fig 1:**
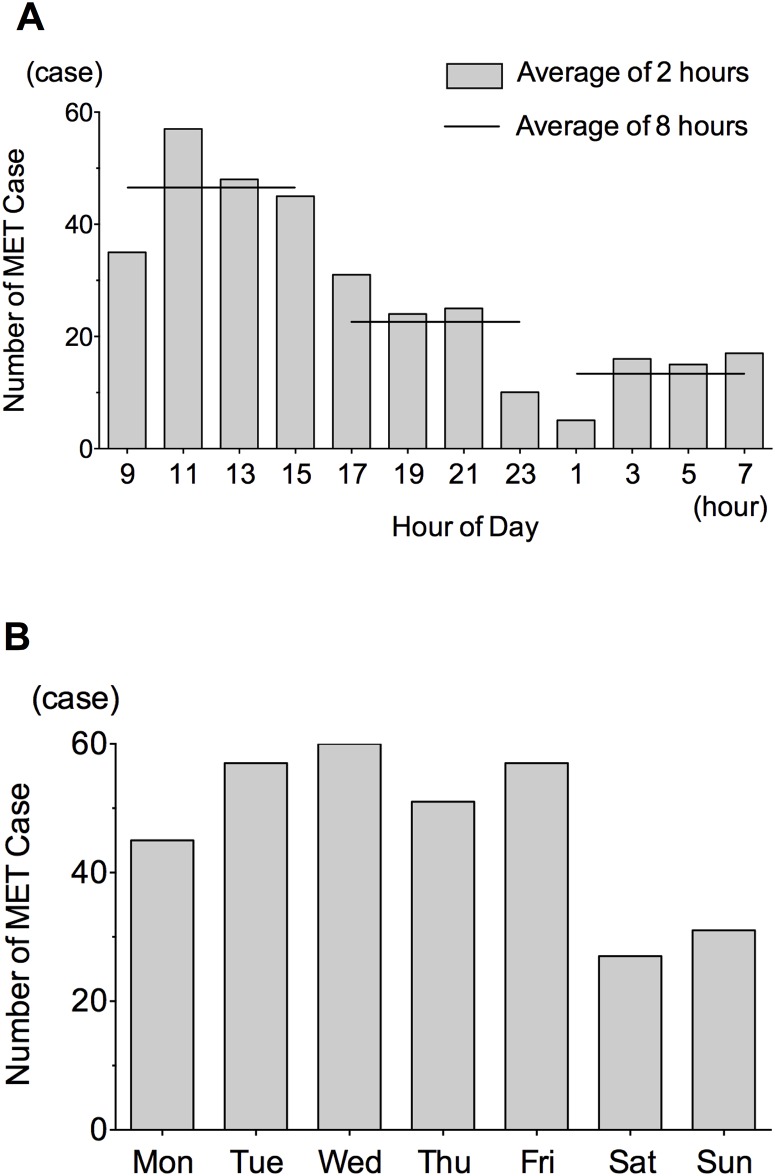
**A.** Number of patients for whom the MET was activated: by hour of day **B.** Number of patients for whom the MET was activated: by day of week.

Of the 328 study subjects, the MET was activated for the largest number during the daytime (9:00–16:59; n = 185); this was followed by the evening (17:00–0:59; n = 90), and finally by the night-time (1:00–8:59; n = 53; Fig 1A). Furthermore, the MET was activated more often on weekdays than at the weekend (Fig 1B). Put another way, the MET was activated less frequently during off-hours.

**Fig 2:**
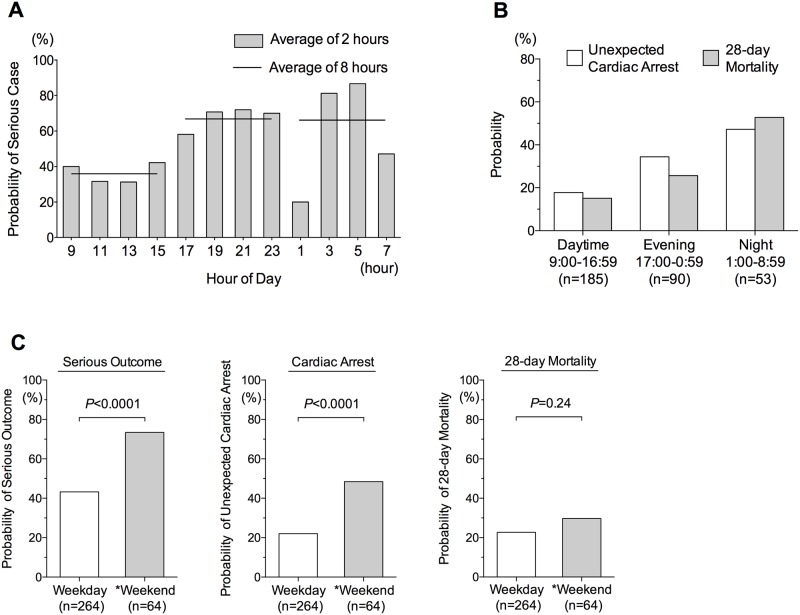
**A.** Probability of serious case: by hour of day **B.** Probability of unexpected cardiac arrest, and 28-day mortality. The probability of unexpected cardiac arrest and 28-day mortality in patients for whom the MET was activated increased from daytime toward night-time (daytime < evening < night-time; *P* < 0.0001 by chi-square test for trend). **C.** Probability of serious outcome, unexpected cardiac arrest, and 28-day mortality: weekday vs weekend. Patients for whom the MET was activated on a weekday had a lower probability of short-term serious outcome, cardiac arrest, and 28-day mortality than those for whom the MET was activated at the weekend (short-term serious outcome and cardiac arrest *P* < 0.0001; 28-day mortality *P* = 0.24 by chi-square test).*We defined “weekend” as weekend and public holiday.

Furthermore, there were significant differences among the timing categories of MET activation in terms of the incidence of short-term serious outcome (Fig 2A), unexpected cardiac arrest, and 28-day mortality (Fig 2B). Specifically, the probability of each adverse outcome increased from daytime toward night-time (daytime < evening < night-time; *P* < 0.0001 by chi-square test for trend; Fig 2B and 2C). Patients for whom the MET was activated on weekdays had a lower probability of short-term serious outcome, cardiac arrest, and 28-day mortality than those for whom the team was activated at the weekend (short-term serious outcome and cardiac arrest: *P* < 0.0001; 28-day mortality: *P* = 0.24; Fig 2C). Thus, the probability of adverse outcomes appeared to increase during off-hours in univariate tests.

**Fig 3:**
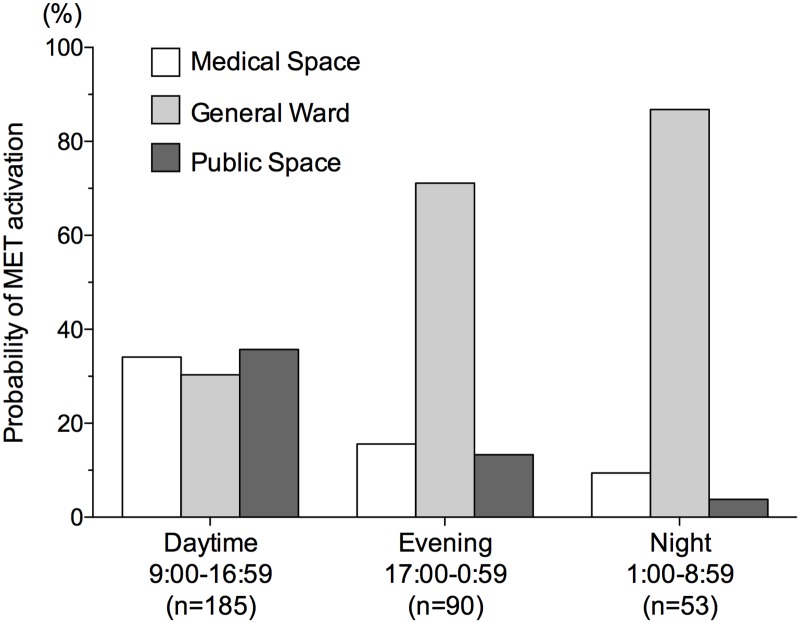
Probability of MET activation: timing and location.

The frequency of MET activation was similar among medical space, general ward, and public space during the daytime, whereas during the evening and night-time, the MET was mostly activated from the general ward (Fig 3).

There were significant differences in baseline characteristics between patients with and without serious outcome (Table 1). Specifically, patients with serious outcome were significantly older and had a higher frequency of inpatient status and cardiovascular diagnosis than patients with non-serious outcome; furthermore, they were more often postoperative or post ICU-discharge patients, and they had a higher incidence of sepsis and chronic organ dysfunction (heart, kidney; Table 1). There were significant differences between the two groups in terms of the timing and location of MET activation (Table 2).

**Table 1:** Baseline characteristics of patients requiring medical emergency team activation.

	Serious group	Non-serious group	
	(n = 161)	(n = 167)	*P-*value
Age in years	65.4 (16.4)	56.3(19.5)	<0.0001
Sex (% men)	60.2	54.5	0.24
Inpatients, n (%)	140 (87.0)	62 (37.1)	<0.0001
Patient category, n (%)			
Medical	46 (28.6)	55 (32.9)	0.39
Surgical	70 (43.5)	62 (37.1)	0.24
Cardiovascular	38 (23.6)	21 (12.6)	0.0093
History			
Postoperative, n (%)	59 (36.6)	25 (15.0)	<0.0001
Post ICU discharge, n (%)	92 (57.1)	23 (13.8)	<0.0001
Sepsis, n (%)	34 (21.2)	13 (7.8)	0.0006
Pre-existing conditions, n (%)			
Chronic heart failure	55 (34.7)	19 (11.4)	<0.0001
Chronic pulmonary disease	28 (17.4)	21 (12.6)	0.23
Chronic kidney disease	20 (12.4)	9 (5.4)	0.032
Chronic liver disease	18 (11.9)	11 (6.6)	0.14
Chronic brain disease	15 (9.3)	18 (10.8)	0.66
Steroid therapy	19 (11.8)	23 (13.8)	0.59
Immune suppression	33 (20.5)	34 (20.4)	0.98
Diabetes	22 (13.7)	14 (8.4)	0.13
Malignancy	56 (34.8)	50 (29.9)	0.35

Serious case: ICU admission after MET activation or death at scene.

Data are median (interquartile range) for continuous variables.

*P*-values were calculated using the Mann–Whitney U test or chi-square test.

**Table 2:** Characteristics, treatments, and outcomes of medical emergency team.

	Serious group	Non-serious group	
	(n = 161)	(n = 167)	*P-*value
Timing of MET activation			
Weekend / public holiday, n (%)	47 (29.2)	17 (10.2)	<0.0001
Hour of day, n (%)			
9:00–16:59	66 (41.0)	119(71.3)	<0.0001
17:00–0:59	60 (37.3)	30 (18.0)	
1:00–8:59	35 (21.7)	18 (10.8)	
Site of incidence, n (%)			<0.0001
Medical space	41 (25.5)	41 (24.6)	
Outpatients department	12 (7.5)	23 (13.8)	
Examination rooms	22 (13.7)	17 (10.2)	
Treatment room	7 (4.3)	1 (0.6)	
General wards	113 (70.2)	53 (31.7)	
Public space	7 (4.3)	73 (43.7)	
Treatments, n (%)			
Intubation	104 (64.6)	21 (12.6)	<0.0001
Ventilation	126 (78.3)	47 (28.1)	<0.0001
Fluid resuscitation	24 (14.9)	13 (7.8)	0.042
Cardiovascular agents	67 (41.6)	14 (8.4)	<0.0001
24-h mortality, n (%)	28 (17.4)	5 (3.0)	<0.0001
7-day mortality, n (%)	47 (29.2)	14 (8.4)	<0.0001
28-day mortality, n (%)	62 (38.5)	17 (10.2)	<0.0001

Examination rooms: Blood collecting room, radiographic examination room, and physiological laboratory room

Treatment rooms: Operating room, endovascular room, dialysis room and delivery room

Medical space: Outpatients department, examination rooms, or treatment rooms

Public spaces: Halls, stairs, lounge or parking lot

Ventilation: bag valve mask or mechanical

Serious case: death or ICU admission after MET activation

Data are median (interquartile range) for continuous variables.

*P*-values were calculated using the Mann–Whitney U test or the chi-square test.

In the primary analysis of the study using multivariate logistic regression, patients for whom the MET was activated in the evening or night-time had significantly higher rates of short-term serious outcome than those for whom the MET was activated during the daytime (evening: adjusted OR = 2. 53, 95% CI = 1.24–5.13, *P* = 0.010; night-time: adjusted OR = 2.45, 95% CI = 1.09–5.50, *P* = 0.030). Furthermore, short-term serious outcome occurred more frequently during the weekend than on weekdays, although this was not a significant result (adjusted OR = 1.76, 95% CI = 0.83–3.72, *P* = 0.14; Table 3). In addition, in patients for whom the MET was activated in public space, short-term serious outcome occurred significantly less often than in patients for whom it was activated in medical space (adjusted OR = 0.19, 95% CI = 0.07–0.54, *P* = 0.0017). Similarly, in patients for whom the MET was activated on the general ward, the incidence of serious outcome was lower than in patients for whom it was activated in medical space, although this difference was not statistically significant (adjusted OR = 0.42, 95% CI = 0.15–1.13, *P* = 0.085; Table 3).

**Table 3:** Association of short-term serious outcome with timing and location of MET activation.

	Odds Ratio (95% CI)	*P-*value
Age, per year	1.02 (1.00–1.04)	0.021
Inpatient	2.93 (0.98–8.76)	0.054
Cardiovascular	1.80 (0.77–4.22)	0.18
Postoperative	1.13 (0.58–2.23)	0.72
Post ICU discharge	3.78 (1.88–7.58)	0.0002
Sepsis	3.35 (1.47–7.64)	0.0041
Chronic heart failure	2.36 (1.05–5.28)	0.037
Chronic kidney disease	0.55 (0.18–1.68)	0.29
Timing of MET activation		
Weekend / public holiday	1.76 (0.83–3.72)	0.14
Hour of day		
9:00–16:59	Reference	
17:00–0:59	2.53 (1.24–5.13)	0.010
1:00–8:59	2.45 (1.09–5.50)	0.030
Site of incidence		
Medical space	Reference	
General ward	0.42 (0.15–1.13)	0.085
Public area	0.19 (0.07–0.54)	0.0017

*P*-values were calculated using multivariate logistic regression analysis, with the following covariates: age, inpatient status, cardiovascular diagnosis, postoperative status, post ICU discharge status, sepsis, chronic heart failure, chronic renal dysfunction, and timing/location of MET call.

Next, in a multivariate logistic regression analysis, we tested for an association between the timing or location of MET activation and unexpected cardiac arrest upon receipt of MET call, as well as with 28-day mortality. The night-time (vs. daytime) and medical space (vs. public space) were significantly associated with higher risks of unexpected cardiac arrest and 28-day mortality (*P* < 0.05, Table 4).

**Table 4:** Association of timing and site of MET activation with unexpected cardiac arrest and 28-day mortality.

	Unexpected cardiac arrest		28-day mortality	
	Adjusted OR (95% CI)	*P-*value	Adjusted OR (95% CI)	*P-*value
Timing of MET call				
Weekend / public holiday	2.15 (1.11–4.14)	0.023	0.76 (0.38–1.54)	0.45
Hour of day				
9:00–16:59	Reference		Reference	
17:00–0:59	1.67 (0.83–3.35)	0.15	1.57 (0.77–3.20)	0.21
1:00–8:59	3.67 (1.66–8.10)	0.0013	6.11 (2.72–13.7)	<0.0001
Site of incidence				
Medical space	Reference		Reference	
General ward	0.43 (0.18–1.06)	0.065	0.886(0.34–2.31)	0.80
Public area	0.28 (0.08–0.98)	0.046	0.184(0.047–0.716)	0.015

*P*-values were calculated using multivariate logistic regression analysis, with the following covariates: age, inpatient status, cardiovascular diagnosis, postoperative status, post ICU discharge status, sepsis, chronic heart failure, chronic renal dysfunction, and timing/location of MET call.

## Discussion

In our study, MET was activated 336 times within 3 years; it yields a frequency of 6.6 /1,000 admission. Chan *et al*. reported that the frequency of MET activation was 2.5–40.3 /1,000 admission. Seventeen studies were systematically reviewed in their report [2]. In MERIT study [20], it was reported that the average of emergency calls was 8.7 /1,000 admission. The frequency of MET activation is strongly affected by the variability of systems in hospitals. The frequency of our MET activation, 6.6 /1,000 admission, is relatively small compared to other studies, however it is not necessarily significant.

In the present study of MET timing and location, we found that the frequency of MET activation was lower during off-hours (evening, night-time, or weekend). Patients for whom the MET was activated in the evening or at night had a significantly higher incidence of short-term serious outcome than those for whom the MET was activated during the daytime; the same was true of patients for whom the MET was activated in medical space: they had a significantly higher incidence of short-term serious outcome than those for whom the MET was activated in public space. A non-significant trend was also found, whereby short-term serious outcome was more frequent in patients for whom the MET was activated in medical space than in those for whom it was activated on a general ward. Furthermore, night-time and medical space were significantly associated with higher risks of unexpected cardiac arrest and 28-day mortality.

In the present study, the incidence of MET activation was higher during the daytime and on weekdays. Previous studies have consistently shown a high frequency of MET activation during the daytime [6, 10] or work hours (daytime on a weekday) [8]. In one study, the MET was activated more often on Mondays [8]; in another, the frequency of MET activation was higher in the morning (6:01–12:00) [9]. Such trends were not identified in the present study. According to previously reported data, nurse workflow, including scheduled rounds for observation or handover, is associated with the frequency of MET activation [10, 11]. In the present study, we could not find similar trends, because we lacked data on routine observation or handover. Nonetheless, we found a very low frequency of MET activation during the 4-hour period after 23:00; this has consistently been observed in previous studies [10, 11]. Since nightshift nurses in our hospital often take turns to have breaks during this 4-hour period, the low incidence of MET activation may be linked with nurse workflow.

Patients for whom the MET was activated during off-hours had a high probability of short-term serious outcome, unexpected cardiac arrest, and 28-day mortality in univariate analysis (Fig 2). The off-hours effect on short-term serious outcome was also observed in the primary analysis using multivariate logistic regression (Table 3; evening and night *P* < 0.05; weekend *P* = 0.091). To the best of our knowledge, the current study was the first to demonstrate an association between the timing of MET activation and short-term serious outcome.

We also found an association of night-time and weekend MET activation with unexpected cardiac arrest (Table 4; night and weekend *P* < 0.05). In accordance with this, Jones *et al*. previously reported a high incidence of in-hospital cardiac arrest during the night-time between 2:00 and 3:00 AM, and between 6:00 and 7:00 AM. They conducted a historical control study to compare the incidence of in-hospital cardiac arrest before and after the introduction of the MET service. They concluded that immediate MET activation might decrease the incidence of cardiac arrests during the night [11].

We found similar frequencies of MET activation in medical space, on general wards, and in public areas during the daytime; in contrast, during the evening and night-time, the MET was more frequently activated in general wards than in medical spaces or public areas. Despite the paucity of data on the relationship between locations and incidence of MET activation, our finding is not surprising, because the outpatient department, examination rooms, and treatment rooms are mostly used during the daytime.

In the present study, patients for whom the MET was activated in a medical space, including examination and treatment rooms, had a significantly increased risk of short-term serious outcome, unexpected cardiac arrest, and 28-day mortality than those for whom the MET was activated in public areas. In addition, there was a higher incidence of serious outcome and unexpected cardiac arrest in medical space than on the general ward, although the trend was not significant. Although there have been few investigations into similar location effects, our findings may be consistent with previous reports emphasizing the importance of procedures and conditions in the examination [21, 22] and treatment rooms [23–25] to the risk of adverse events.

We show that medical space, weekend, evening and nighttime are risk factors for serious outcomes of patients. According to this result, we could add two interpretations. First, it is important to properly prepare MET members for a patient who is under these risk factors. Identifying risk factors may be the key to improving clinical outcome in patients for whom the MET is activated. Specifically, recognizing risk and developing specific countermeasures may improve the afferent limbs of the MET system, including early crisis detection and rapid response triggering, as well as the efferent limbs, such as rapid and better treatment in anticipation of possible risk [1, 26]. If we increase healthcare providers’ awareness of the risk during off-hours and in medical spaces, they may recognize medical emergencies and activate the MET more quickly. Of particular importance are healthcare workers in medical spaces, as well as the furtherance of specialized strategies or protocols for off-hour or medical space MET activation. This may in turn improve clinical outcomes in patients for whom the MET is activated. And second, by figuring out the reason why these risk factors led to serious outcomes, our MET activities can be improved and it could be possible to improve patient outcomes. In the present study, we analyzed only patients for whom MET activation was requested, and therefore it is difficult to evaluate how properly MET was activated in weekend, evening and nighttime. This is the significant limitation of our study. It is considered to be a task for the next study analyzing how many patients are deteriorating and how properly MET are activated. It is considered important to investing the relation between MET activations and our hospital local situation (the number of caregivers, observation frequency, monitoring system, and so on).

The present study was the first to show an association between worse clinical outcomes and (1) timing and (2) location of MET activation; this was the strength of the study. However, these results were based on data from single center, and the sample was not large; these constitute the limitations of the study. Further studies using a larger sample from multiple centers may strengthen our study results.

## Conclusions

Patients for whom the MET was activated had higher rates of short-term serious outcome in the evening, at night-time, and in medical spaces. If we take measures against these differences by increasing the requesters’ and the MET’s awareness of the risk factors, we may improve the afferent and efferent limbs of the MET system, as well as clinical outcomes.

## Supporting Information

S1 File MET activation data file. (XLSX)

## Abbreviations

CI: confidence interval
ICU: intensive care unit
MET: Medical emergency team
OR: odds ratios
